# Breath rate of passerines across an urbanization gradient supports the pace‐of‐life hypothesis and suggests diet‐mediated responses to handling stress

**DOI:** 10.1002/ece3.4460

**Published:** 2018-08-29

**Authors:** Dan Liang, Chao He, Xu Luo, Yang Liu, Eben Goodale, Emilio Pagani‐Núñez

**Affiliations:** ^1^ State Key Laboratory of Biocontrol Department of Ecology/School of Life Sciences Sun Yat‐sen University Guangzhou China; ^2^ Guangxi Key Laboratory of Forest Ecology and Conservation College of Forestry Guangxi University Nanning China; ^3^ State Forestry Administration Key Laboratory of Biodiversity Conservation in Southwest China Southwest Forestry University Kunming China

**Keywords:** acute stress responses, anthropogenic disturbance, diet specialization, handling stress, trophic guild, urban–rural differences

## Abstract

The pace‐of‐life hypothesis predicts no impact of urbanization on stress responses. Accordingly, several studies have been inconsistent in showing differences in breath rate (BR), a proxy of acute stress responses to handling in passerines, between rural and urban areas. However, this evidence is limited to a single bird species and a limited geographic region (SW Europe). No study addressed whether this pattern is also apparent in other species or regions, such as in tropical environments, or whether it is dependent on the level of diet specialization, given that diet restriction and change influence stress responses. Here, we tested whether there were differences in BR between habitats and diet groups using eight highly diverse passerine assemblages experiencing different levels of anthropogenic disturbance (i.e., natural, rural, and urban locations) in SW China. We predicted that insectivores and herbivores (frugivores, nectarivores, and seed‐eating species) would show higher BR than omnivores. We also predicted no differences in BR among habitat types. BR was a moderately repeatable trait, which showed a negative relationship with body mass and a positive relationship with the time of the day. We also recorded a relatively strong phylogenetic bias in the expression of this trait. Confirming our predictions, our results showed no differences in BR among natural, rural, and urban locations. Similarly, within species, there were no differences in BR between rural and urban locations. However, we also found that herbivores showed higher BR than omnivores. Overall, our results provide support to the pace‐of‐life hypothesis, but suggest acute stress responses can be diet‐mediated, which may help to explain the marked decline of specialized trophic guilds around the world in response to anthropogenic disturbance.

## 1 INTRODUCTION

Vertebrates display stress responses to cope with harmful and unpredictable stimuli (i.e., stressors) (Romero, 2004). Across their evolution, natural stressors, to which vertebrates have had time to adapt, have shaped these responses. However, since the industrial revolution, anthropogenic disturbance has become a key source of stress for wildlife (Wingfield, 2013). During the last decades, such stressors have become widespread due to a fast and exponential increase in anthropogenic disturbance around the world (Hendry, Gotanda, & Svensson, 2017; Vitousek, Mooney, Lubchenco, & Melillo, 1997). These impacts are particularly evident as a result of the progressive transformation of natural habitats, which includes drastic changes in abiotic factors, habitat structure, food quality and quantity, and human population density and activity. This increase in anthropogenic disturbance is known to drive a homogenization effect on biodiversity (McKinney, 2006) and to select certain phenotypes able to cope with these new stressors (Alberti, Marzluff, & Hunt, 2017).

Yet, not all species respond in the same way to anthropogenic disturbance. Previous research has suggested that generalist species are less sensitive to anthropogenic disturbance than specialists due to their higher physiological tolerance (Bonier, Martin, & Wingfield, 2007). Hence, as natural habitats are urbanized, some species are lost (McKinney, 2008), whereas others display physiological responses to cope with these novel environments (Chevin, Lande, & Mace, 2010; see also Isaksson, 2015). Inspired by previous research on rural–urban differences, the pace‐of‐life hypothesis suggests that individuals colonizing urban environments show a particular suite of life history traits (i.e., high investment in self‐maintenance and low investment in reproduction), but also predicts no change in stress responses (Sepp, McGraw, Kaasik, & Giraudeau, 2018). Accordingly, most research on the topic has shown that species display limited or no changes in stress responses when exposed to anthropogenic disturbance (Bonier, 2012; Chavezzichinelli et al., 2010; Hudin et al., 2018; Sepp et al., 2018). This has raised the question whether urban areas are not particularly stressful habitats, or at least similarly stressful compared to natural environments, for the species able to colonize them.

Breath rate (BR), measured as the number of breaths per time unit, namely the number of breast movements indicative of inspirations and expirations, is an indicator of acute stress responses to handling in passerines (Carere & van Oers, 2004). Stress responses are usually quantified through corticosterone levels in blood (Fokidis, Orchinik, & Deviche, 2009), feathers (Bortolotti, Marchant, Blas, & German, 2008), and feces (Casas et al., 2016), but these approaches are time‐consuming and costly. In contrast, measuring BR is an inexpensive and direct method that can be used as a rapid assessment tool. Although no study has established a direct link between corticosterone levels and BR, there is a clear link between BR and a brain center that plays a key role in generalized alertness, attention, and stress (Yackle et al., 2017; see also Noble, Goolsby, Garraway, Martin, & Hochman, 2017). Moreover, behavioral traits can show higher individual repeatability than hormone levels, which have been shown to be extremely variable (Holtmann, Lagisz, & Nakagawa, 2017; Weaver, Gao, & McGraw, 2018). However, to completely understand BR, it is necessary to control for several related factors. Body mass strongly influences metabolism (Speakman, 2005) and likely determines interspecific differences in BR, as it determines basal metabolic rates (Londoño, Chappell, Castañeda, Jankowski, & Robinson, 2015). Stress responses also show considerable daily and seasonal temporal variability (Romero & Remage‐Healey, 2000).

Previous research has shown inconsistent results when assessing changes in BR in response to urbanization, with different studies showing increased BR toward more urbanized areas (Charmantier, Demeyrier, Lambrechts, Perret, & Grégoire, 2017; Torné‐Noguera, Pagani‐Núñez, & Senar, 2014) or no significant differences in stress responses to handling between rural and urban birds (Senar et al., 2017). However, this evidence is limited to a single species (great tits *Parus major*) and to a limited geographic region (SW Europe). Additional research using more species in different habitats or regions is therefore necessary to confirm these patterns. This knowledge gap on the consequences of urbanization is particularly evident in Southeast Asia, one of the most diverse and densely populated areas of the world (Chace & Walsh, 2006), and a region in which urbanization rates are currently very high (Seto, Güneralp, & Hutyra, 2012). Here, we analyzed for the first time BR variation at a community level, specifically measuring BR in eight subtropical passerine assemblages in natural, rural, and urban locations that strongly differ in species composition.

Additionally, diet has a relevant role in determining species capacity to colonize urban areas (Coogan, Raubenheimer, Zantis, & Machovsky‐Capuska, 2018), and diet restriction and change are known to induce increased stress responses (Skinner et al., 2016; Will et al., 2015). Changes in diet composition have been described to impact fatty acid composition and oxidative stress across urbanization gradients (Isaksson, Andersson, Nord, von Post, & Wang, 2017). We thus hypothesized that while omnivores would be able to handle stress associated with anthropogenic disturbance, more specialized groups, such as herbivores and insectivores, would be more affected by these stressors. We label this idea as the “diet‐induced stress response hypothesis.” We predicted that birds with herbivore and insectivore diets would show relatively higher BR at handling than omnivores.

On the other hand, although the pace‐of‐life hypothesis predicts no impact of urbanization on stress responses, it also suggests substantial behavioral changes that may result in decreased fear (or increased risk taking) toward humans (Sepp et al., 2018; see also Griffin, Netto, & Peneaux, 2017). Thus, acknowledging that urban species may be well habituated to anthropogenic disturbance, and given that BR is collected while handling the birds, we predicted that urban species would display decreased BR when compared with species inhabiting rural and natural habitats. We labeled this alternative hypothesis as the “habituation‐induced stress response hypothesis.”

In summary, we tested whether diet specialization (“diet‐induced stress response hypothesis”) or behavioral habituation to human disturbance (“habituation‐induced stress response hypothesis”) are the main drivers of variability in acute stress responses of passerines.

## 2 MATERIALS AND METHODS

### 2.1 Study locations

**Figure 1:**
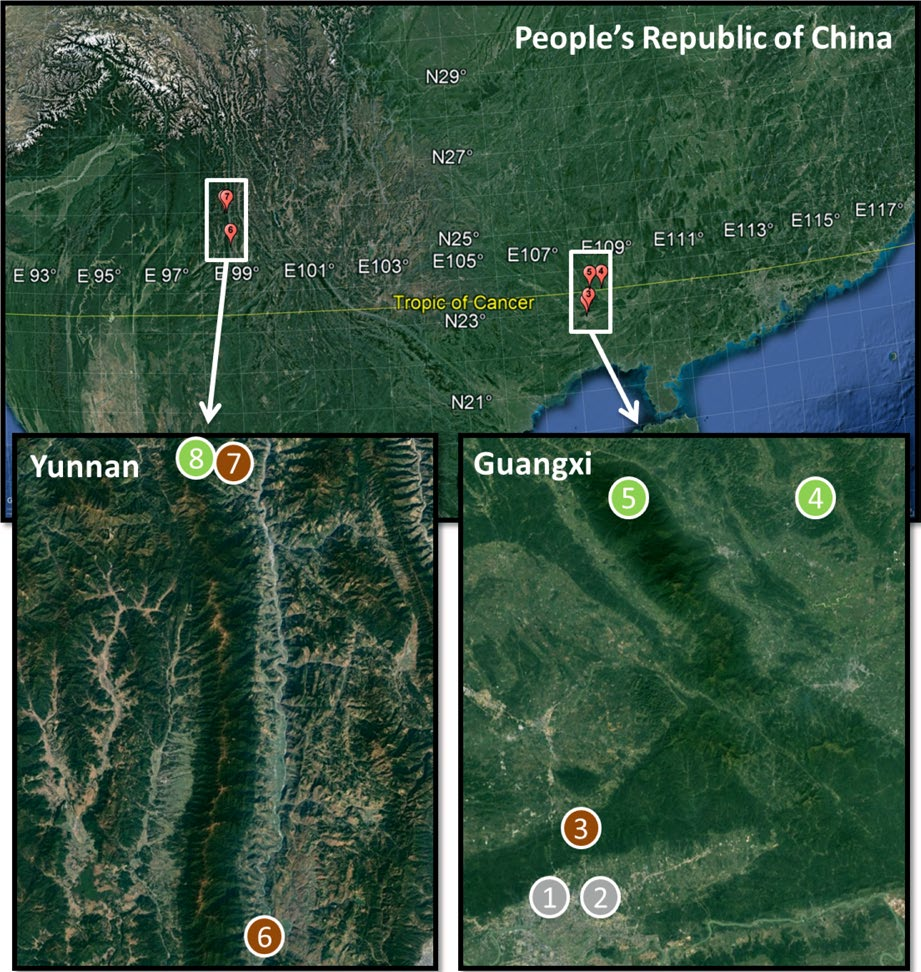
Maps showing the position of our study locations at Yunnan Province and Guangxi Zhuang Autonomous Region. In the upper part, there is an overview map of the P.R. China, and in the lower part, we zoomed, at the same scale, on the areas of interest (see Supporting Information Appendix S1 for detailed satellite images from each location). Our study locations are marked in gray (urban), brown (rural), or green (natural)

We banded a total of 963 individuals from 114 passerine bird species using mist nets in eight locations at Guangxi Zhuang Autonomous Region and Yunnan Province (SW China) from March 2016 to February 2017 (Table 1; Figure 1). We chose these locations because they show enough variation in species composition, elevation, and habitat disturbance to effectively test our predictions. We also recaptured 57 individuals, which we measured again (see Section 2.2 for detailed information). These data were used to compute repeatability of BR. We followed a constant banding effort scheme in four locations located in Guangxi (Mango Fields, Medicinal Botanical Garden, Gaofeng Forest, and Longshan NR). These locations are located at low elevation (range: 82–234 m; Table 1; Figure 1; Supporting Information Appendix S1). We visited each of these locations at least one time every 2 months. Mist nets were set for two consecutive days at dawn and monitored for 6 hr each day. Nets were checked every hour. Fieldwork was interrupted in July and August due to unusually high temperatures (up to 50°C), which implied a risk for birds' lives and anomalous conditions that could bias our results. The other four locations were located at high elevation (range: 734–2,377 m) in Damingshan (Guangxi) and three locations at Gaoligong Mountains (Yunnan) (Saige Valley, Luzhang, and Yaojiaping NR) (Table 1; Figure 1; Supporting Information Appendix S1). In these remote locations, we followed a more intensive banding procedure due to time constraints (see Section 3 for a comparison of BR values between these two protocols). Mist netting was performed from dawn to dusk during several consecutive days (from 1 week to 1 month) during the dry season (from October to February). Both areas are characterized as “subtropical” in the Koppen climate classification. Here, there are two seasons, which are usually referred to as rainy (May–October) and dry (November–April) (Zheng, 2000).

**Table 1:** Characteristics of our study locations

Locality	Coordinates	Region	Habitat type	Elevation (m)	Species richness	*N* individuals caught	Sampling effort (*N* hours)	Abundance index (*N* ind./hour)
1. Mango Fields	22°51′09.14″–108°17′21.41″	Guangxi	Urban	82	31	153	60	2.55
2. MedBotGard	22°51′3.85″–108°22′38.66″	Guangxi	Urban	112	22	117	60	1.95
3. Gaofeng	22°57′4.33″–108°30′27.78″	Guangxi	Rural	234	23	98	60	1.63
4. Longshan	23°29′24.28″–108°45′25.14″	Guangxi	Natural	113	24	98	48	2.04
5. Damingshan	23°29′50.89″–108°26′16.69″	Guangxi	Natural	1243	15	99	78	1.27
6. Luzhang	25°57′41.00″–98°46′30.61″	Yunnan	Rural	1781	26	67	78	0.86
7. Saige Valley	25°7′38.57″–98°51′22.00″	Yunnan	Rural	734	27	112	48	2.33
8. Yaojiaping	25°58′16.00″––98°42′37.70″	Yunnan	Natural	2377	24	223	102	2.19

Notes

Habitat type was categorized according to anthropogenic disturbance. See Supporting Information Appendix S1 for satellite images from each location.

We categorized each location according to the level of anthropogenic disturbance using a common nomenclature, that is, natural, rural, and urban locations (e.g., Marzluff, Bowman, & Donnelly, 2001). We used the level of human occupancy and activity as main criteria to categorize locations in relation to anthropogenic disturbance (Table 1; Supporting Information Appendix S1). Natural locations (*N* = 3) were protected forests with almost no human occupancy and relatively low human activity (i.e., low anthropogenic disturbance). Rural locations (*N* = 3) were crop and forest areas with low human occupancy and moderate human activity (i.e., moderate anthropogenic disturbance). Urban locations (*N* = 2) were located in Nanning, an urban area with very high human activity (i.e., high anthropogenic disturbance).

### 2.2 Data collection

We collected data on BR and body mass from all individuals. Once the bird was extracted from the net, we kept it in a bag for at least 5 min until we took the bird out. Although not all individuals spent the same time in the nets, all individuals were given a similar period of time to rest inside the cloth bag, so that we obtained a standard and comparable measure from all individuals. We noted the time (number of hours since dawn), season (rainy or dry), and species identity. We then measured BR following Torné‐Noguera et al. (2014). We placed the bird on its back with its head held between the index and central fingers and tarsi held with the other hand. In doing so, birds could not see the face of the person holding it. We then counted the number of breast movements over 30 s. While recording BR, we kept our movements and speech to a minimum. Finally, we measured the body mass using a digital balance to the nearest 0.1 g. We only measured healthy birds and observed no harm to them by the capture and measuring process.

### 2.3 Species categorization

Birds were classified according to their diet using EltonTraits 1.0 database (Wilman et al., 2014). Diet categories were assigned depending on the most common food types ingested by each species and included omnivore, plant seed eating, frugivore–nectarivore or insectivore. We pooled frugivore–nectarivore diets and plant seed‐eating species as herbivores because their sample sizes were low. In relation to migratory status, we classified each species as migrant or resident according to the descriptions provided by the Handbook of the Birds of the World (del Hoyo, Elliott, Sargatal, Christie, & de Juana, 2018). See Supporting Information Appendix S2 for a list of species' numbers per location, including the information on habitat and diet groups.

### 2.4 Statistical analyses

We evaluated the influence of temporal variation on BR. To do this, we computed a linear mixed‐effects model (LME) using nlme v3.1‐131 (Pinheiro, Bates, DebRoy, & Sarkar, 2017), with log‐transformed BR individual values as the dependent variable, season (rainy or dry) as the fixed factor, and time of day (number of hours since dawn) (g) as a covariate. We included species identity and location as random factors. We only used one measurement per individual (the first when there was more than one) and excluded species with less than two individuals. Thus, we reduced our sample to 879 individuals from 69 species. We extracted residuals from this model to carry out further analyses. We also assessed whether the two different protocols influenced our results after controlling for these factors. We used an analysis of variance with standardized BR values (i.e., residuals from the first model) as the dependent variable and field protocol (constant effort vs. intensive banding) as the fixed factor. Additionally, sex and age factors may influence BR (Markó et al., 2013; see also Holtmann et al., 2017), but our inability to determine sex and age of all individuals prevented us from controlling for these factors. Regardless, high overlap and similar variability in BR values between identifiable sex and age classes suggest that overall these factors had a small effect on our results (Supporting Information Appendix S3).

We computed individual repeatability of BR, using rptR v0.9.2 (Stoffel, Nakagawa, & Schielzeth, 2017), as a way to determine its temporal consistency (i.e., whether BR was consistent across time and whether relevant individual differences existed). We computed repeatability using individual identity as the grouping factor and included time of the day as a covariate and season as the fixed factor (given that these individuals were recaptured at different moments of the day and the year). We used two measurements per individual and only included species with at least two individuals. As a result, we considered 50 individuals from 11 species.

Species turnover was very high (only 19 of 69 species were present in more than one location), and species composition was, therefore, unique in each location (Supporting Information Appendix S2). Although there was considerable intraspecific variability, trait variation across localities was mostly due to species turnover rather than to intraspecific variation (following Lepš, de Bello, Šmilauer, & Doležal, 2011). Thus, we used BR measurements from individuals in the location where a species had the largest numbers and excluded the rest of individuals of that species in other locations (Supporting Information Appendix S2). We had to exclude “Damingshan” as a location because it had very small sample size after this procedure (*N *=* *2). In doing so, we were able to carry out phylogenetically controlled analyses, given that species could not be repeated in more than one location.

We quantified the phylogenetic signals (Pagel's λ, Pagel, 1999) of BR, body mass, and their regression residuals using *phytools* v0.6 (Revell, 2012). We downloaded 10,000 trees of 69 species from avian “tree of life” (Jetz, Thomas, Joy, Hartmann, & Mooers, 2012) and generated a maximum clade credibility tree using TreeAnnotator v1.8.2 of the BEAST software (Drummond & Rambaut, 2007).

To assess the relative importance of habitat type and diet on interspecific variation of BR, we used Bayesian phylogenetic mixed models in the package MCMCglmm (Hadfield, 2010). We included body mass, migratory status, and elevation in this model because these factors may influence interspecific variation in stress responses (see, e.g., Londoño et al., 2015). We ran 750,000 iterations and fixed a thinning interval of 300 and a burn‐in of 75,000 with a Gaussian distribution. We ran the models three times and checked that the Gelman–Rubin statistic was less than 1.02 (Gelman & Rubin, 1992). Visual inspection of the MCMC trace plots revealed a low degree of autocorrelation and appropriate model convergence (Hadfield, 2010). We used average standardized BR values as the response variable. We included body mass (g), diet (herbivore, insectivore, or omnivore), habitat (natural, rural, or urban), elevation (m), and migratory status (resident or migrant) as fixed variables. Body mass was log‐transformed to improve model convergence. We dummy‐coded diet and habitat groups as three‐level variables, and each of the levels of these two groups was manually modified as references in separate models. Finally, we ranked candidate models according to their DIC values (deviance information criterion) (Spiegelhalter, Best, Carlin, & Van Der Linde, 2002). The model with the lowest DIC value was considered the best and is shown in the Section 3 (Supporting Information Appendix S4).

We finally evaluated the effect of habitat type on intraspecific variation in BR. To do this, we compared BR between rural and urban habitats, but within species. We only included species with more than five individuals in each location, and we combined our two urban locations to increase the sample size. As a result, we included rufous‐capped babbler *Stachyris ruficeps* (insectivore), common tailorbird *Orthotomus sutorius* (insectivore), red‐whiskered bulbul *Pycnonotus jocosus* (omnivore) and scaly‐breasted munia *Lonchura punctulata* (herbivore). We used an ANCOVA approach, with standardized BR of individuals as the response variable, habitat as the fixed variable, and log‐transformed body mass as a covariate. We ran a separate model for each species.

All the analyses were carried out in R software v3.4 (R Core Team 2017). We used *ggmap* (Kahle & Wickham, 2013) and *ggplot2* (Wickham, 2009) packages to create the figures. Standard deviance (±*SD*) is provided when available.

## 3 RESULTS

### 3.1 BR characterization

**Figure 2:**
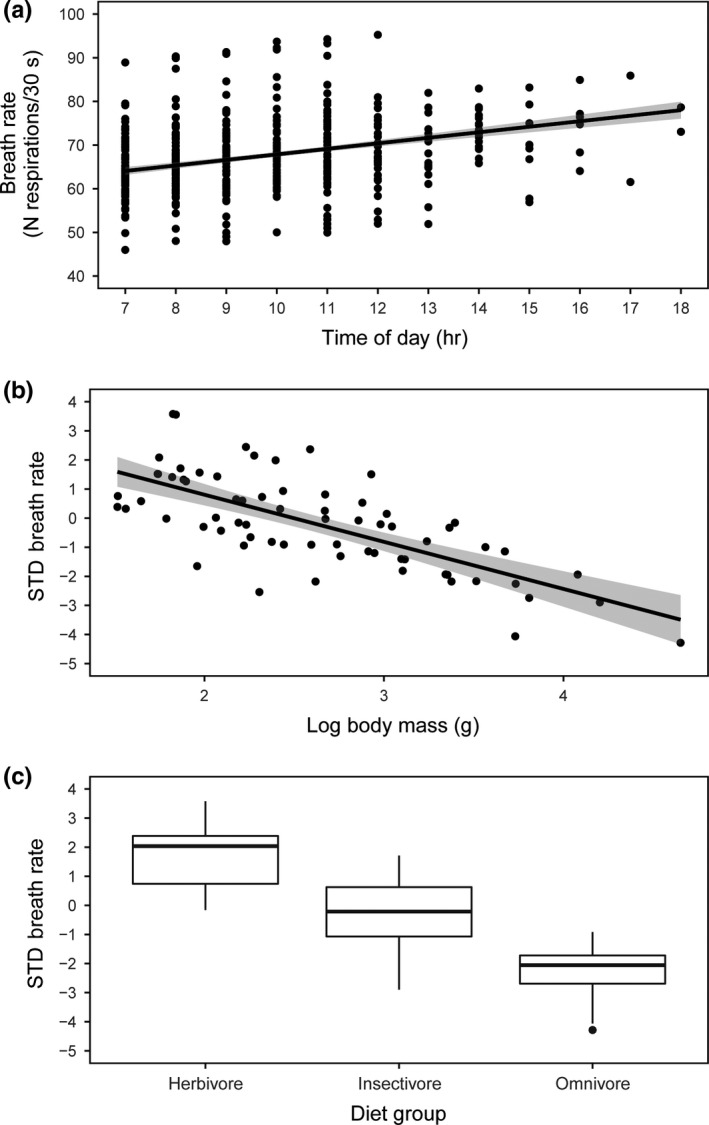
(a) Relationship between time of the day and breath rate (BR) of all the species considered in this study (*N *=* *879 individuals from 69 species). We used raw data as in the corresponding model. (b) Relationship between body mass and BR (*N *=* *69 species). Finally, we show (c) differences in BR among diet groups. In (b) and (c), we used the residuals of the first model, that is, data standardized by temporal variation, as in the corresponding models. In all cases, we plotted the model‐predicted values, controlling for phylogenetic relationships between species and the other factors, to improve the appearance and smoothness of the plots

We found that season had no significant effect on BR (estimate: −0.42 ± 1.579, *t *= −0.266, *p *=* *0.790), while time of day (estimate: 0.98 ± 0.218, *t *=* *4.48, *p *<* *0.001) correlated positively with BR (i.e., later times of the day had higher BR) (Figure 2a). This first model, considering both fixed and random factors, explained almost half variation in BR (conditional R_GLMM^2^ = 0.431). Sampling protocol had no significant effect on BR (constant effort: 0.157 ± 11.200, *N *=* *423 vs. intensive banding: −0.145 ± 11.095, *N *=* *456) (*F *=* *0.162, *p *=* *0.687). Repeatability of BR was moderate after controlling for season and time variability (*R *=* *0.56 ± 0.10, 95% CI = 0.32–0.72; *logLik* = −382.31, *D *=* *19.1, *p *<* *0.01).

### 3.2 Interspecific BR variation

**Figure 3:**
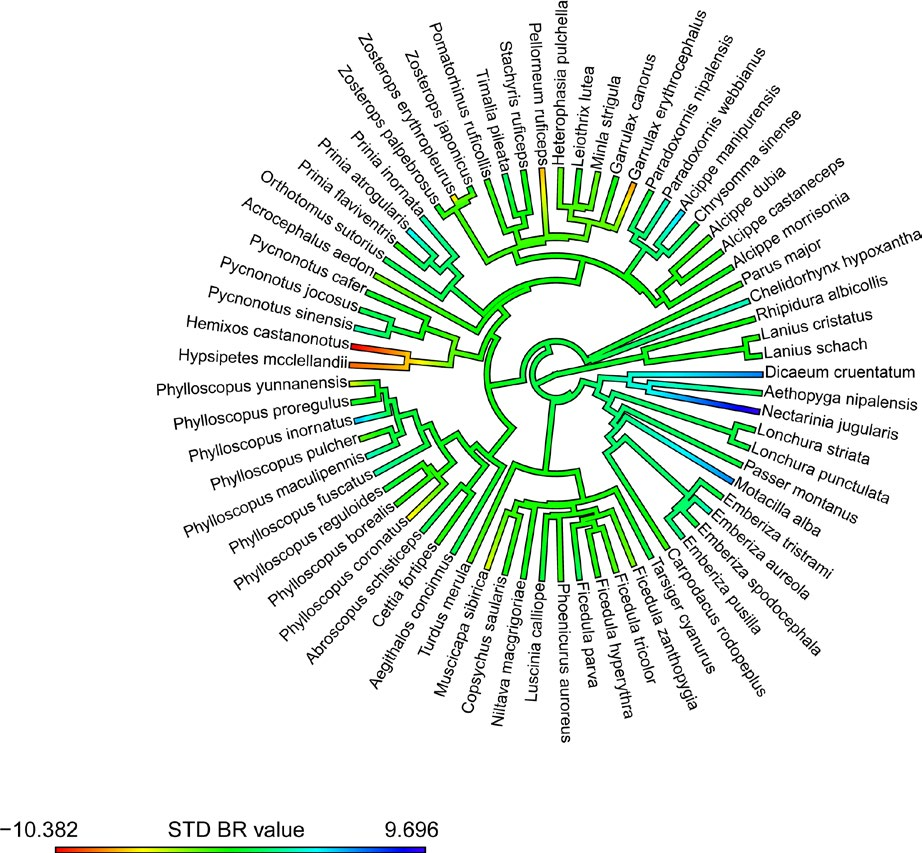
Phylogenetic tree showing breath rate (BR) mean values across the songbird species for which we computed species means (*N *=* *69 species). These values were standardized by temporal variation before computing them

We recorded a very strong phylogenetic signal for body mass (λ* *= 1, *logL* = −276.21), a relatively strong phylogenetic signal for BR (λ* *= 0.63, *logL* = −176.99) (Figure 3), and a moderate phylogenetic signal for their residuals (λ* *= 0.50, *logL* = −172.18). Body mass was negatively correlated with BR (Table 2; Figure 2b).

**Table 2:** Breath rate variation among habitat types (natural, rural, or urban) and diet groups (herbivores, insectivores, and omnivores), controlling for phylogeny, body mass (log‐transformed), migratory status (migrant or resident), and elevation

	β	*SE*	l‐95% CI	U‐95% CI	*pMCMC*
Intercept	6.092	1.938	2.293	9.874	0.013
Random effects					
Phylogeny	2.622	1.338	0.0003	12.8000	
Fixed effects					
Log (body mass)	−1.333	0.571	−2.453	−0.195	0.030
Diet (herbivore–insectivore)	−1.649	1.126	−3.856	0.805	0.178
Diet (herbivore–omnivore)	−3.486	1.435	−6.299	−0.416	0.023
Migration (resident–migrant)	−1.470	0.878	−3.191	0.434	0.108
Habitat (rural–urban)	−0.671	1.058	−2.744	1.457	0.554
Habitat (rural–natural)	−0.194	1.208	−2.561	2.119	0.892
Elevation	−0.0004	0.0005	−0.0013	0.0007	0.477

We did not find significant differences in BR among natural, rural, and urban locations. However, BR values differed among diet groups. The best model indicated that herbivores showed higher BR than omnivores, while herbivores showed no differences with insectivores, which were in an intermediary position (Table 2; Figure 2c). Similarly, elevation and migratory status did not have significant effects on BR (Table 2).

### 3.3 Intraspecific BR variation

Within species, we found no evidence of significant differences in BR between urban and rural habitats (Table 3) and did not find significant relationships between BR and body mass (Table 3). Finally, there were no significant effects of the interaction between body mass and habitat on intraspecific BR variation (Table 3).

**Table 3:** The effects of body mass and habitat on intraspecific BR variation of four species

	CT		RCB		SBM		RWB	
	*F*	*p*	*F*	*p*	*F*	*p*	*F*	*p*
Habitat (rural–urban)	0.289	0.596	0.105	0.751	0.663	0.520	0.001	0.970
Log (body mass)	0.463	0.503	0.655	0.432	0.749	0.391	3.525	0.065
Habitat: log (body mass)	0.352	0.559	0.009	0.924	1.141	0.291	1.393	0.240

CT: common tailorbird; RCB: rufous‐capped babbler; SBM: scaly‐breasted munia; RWB: red‐whiskered bulbul.

## 4 DISCUSSION

### 4.1 BR of tropical passerines support the pace‐of‐life hypothesis

The impact of anthropogenic disturbance is increasingly evident around the world (Seto et al., 2012), but our knowledge on how it impacts species phenotypes and genotypes is still rather limited (Johnson & Munshi‐South, 2017). Previous research has shown that species usually show no or limited responses to anthropogenic disturbance (Bonier, 2012; Chavezzichinelli et al., 2010; Hudin et al., 2018; Sepp et al., 2018). In line with these results, the pace‐of‐life hypothesis predicts no differences in stress responses between urban and rural habitats (Sepp et al., 2018), but previous studies have been inconsistent in confirming this point with regard to BR variation, a proxy of acute stress responses to handling (Charmantier et al., 2017; Senar et al., 2017; Torné‐Noguera et al., 2014). Our results, using hundreds of individuals from over a hundred species of passerines, demonstrate that there were no differences in BR, both between and within species, among natural, rural, and urban locations. Therefore, we expand previous findings to a broader geographic area and confirm this hypothesis at the community level.

Superficially, it would seem reasonable that the positive correlation between BR and time of day might be due to an increase in body temperature with ambient temperature. However, body temperature and BR have been shown to be uncorrelated (Carere & van Oers, 2004). An alternative mechanism could be that hormonal changes lead to increasing organismal responsiveness, in addition to a decrease in energy reserves, across the day (Romero & Remage‐Healey, 2000; Weaver et al., 2018). Further research would be needed to ascertain the exact drivers of this increase in BR across the day. We also recorded a significant negative relationship between body mass and BR, although this pattern was not consistent within species. This suggests that while body mass is an accurate predictor of BR across species, in a similar fashion to the strong and positive relationship between body mass and metabolic rate (Londoño et al., 2015), it may be not so relevant to explain within‐species, or between‐individual, variation.

### 4.2 BR of tropical passerines suggest diet‐mediated stress responses

BR variation may be influenced by phylogenetic or biogeographic inertia like some other traits have been shown to be (e.g., Blomberg & Garland, 2002), so that certain species might show unusually high or low BR. Yet, we controlled our analysis for phylogenetic relatedness and found that herbivores showed higher BR at handling than omnivores. We found support, therefore, for the “diet‐mediated acute stress response hypothesis.” Previous research has shown that the strongest long‐term declines in response to fragmentation of natural habitats mainly corresponded to insectivores and large frugivores, while plant seed‐eating species show higher resilience to anthropogenic disturbance than other guilds (Bregman, Sekercioglu, & Tobias, 2014; Bregman et al., 2016). On the other hand, another study has shown that nectarivores and plant seed eaters seem to be particularly constrained in tropical agrosystems (Tscharntke et al., 2008). Our results support the view that plant seed‐eating species may cope with these disturbances by displaying increased stress responses, which may facilitate their persistence in anthropogenic habitats.

On the other hand, while increased BR may evidently signal increased stress, they could also be seen as a condition *sine qua non* to thrive for certain species. In other words, for certain guilds or species, displaying marked acute stress responses could be necessary to thrive in these environments. However, the lack of changes in BR at handling of an herbivore species (scaly‐breasted munia) in response to increasing urbanization also suggests that this pattern may be intrinsically linked to herbivore ecology, rather than being a specific response to anthropogenic disturbance. Finally, decreased stress responses may be apparent in species particularly well suited to live in rural or urban areas (Partecke, Schwabl, & Gwinner, 2006). Yet, our results do not support this has happened in the bird communities we studied. We may just speculate that relatively low BR of omnivores in this area may be linked to a broader environmental tolerance than more specialized guilds (Bonier et al., 2007; Devictor, Julliard, & Jiguet, 2008; but see Attum, Eason, Cobbs, & Baha El Din, 2006).

Finally, urban individuals may show behavioral differences with nonurban individuals (i.e., bold personality, reduced antipredator behavior, and decreased fear to humans) (Charmantier et al., 2017; Griffin et al., 2017; Lapiedra, Chejanovski, & Kolbe, 2017; Samia, Nakagawa, Nomura, Rangel, & Blumstein, 2015; Sepp et al., 2018; Sol et al., 2018; see also Atwell et al., 2012). However, we found no support to the “habituation‐induced stress response hypothesis.” Species inhabiting natural, rural, and urban locations, and individuals of the same species in rural and urban locations, showed similar BR at handling.

## 5 CONCLUSION

Tropical and subtropical environments are characterized by a striking biodiversity. This great variety of species is of special interest from many perspectives, and research carried out in this area may produce divergent results compared to studies carried out in temperate regions (Stutchbury & Morton, 2001). In this study, consistent with the pace‐of‐life hypothesis, we found that rural and urban areas can harbor a rich and relatively healthy diversity of wildlife fairly resilient to anthropogenic disturbance (Acevedo‐Whitehouse & Duffus, 2009; Oliveira Hagen, Hagen, Ibáñez‐Álamo, Petchey, & Evans, 2017; Pagani‐Núñez, He, Wu, Peabotuwage, & Goodale, 2017). Yet, we also found that herbivores showed higher BR than omnivores, which suggests that stress responses can be diet‐mediated. Although we were not able to determine the underlying mechanism for this result here, further research should assess whether nutritional (Machovsky‐Capuska, Senior, Simpson, & Raubenheimer, 2016) or niche constraints (i.e., decreased niche width or increased niche overlap) drive this increased BR of herbivores.

## CONFLICT OF INTEREST

None declared.

## AUTHOR CONTRIBUTIONS

EPN conceived the study; CH, DL, and EPN collected the data; DL and EPN analyzed the data and wrote the manuscript; XL, LY, and EG provided advice with statistical analyses and writing. All authors contributed critically to the drafts and gave the final approval for publication.

## DATA ACCESSIBILITY

Data is provided as Supporting Information Appendix S2.
