# Supplementary material for: Breath rate of passerines across an urbanization gradient supports the pace‐of‐life hypothesis and suggests diet‐mediated responses to handling stress
Source: Ecol Evol. 2018 Aug 29;8(18):9526–35. doi: 10.1002/ece3.4460 (PMC6194294; doi:10.1002/ece3.4460)
Supplement: Supplementary file 1 [file ECE3-8-9526-s001.pdf]

Appendix 1 List of satellite images of our study locations, which were classified as urban, rural and natural (forests) habitats.

Urban locations

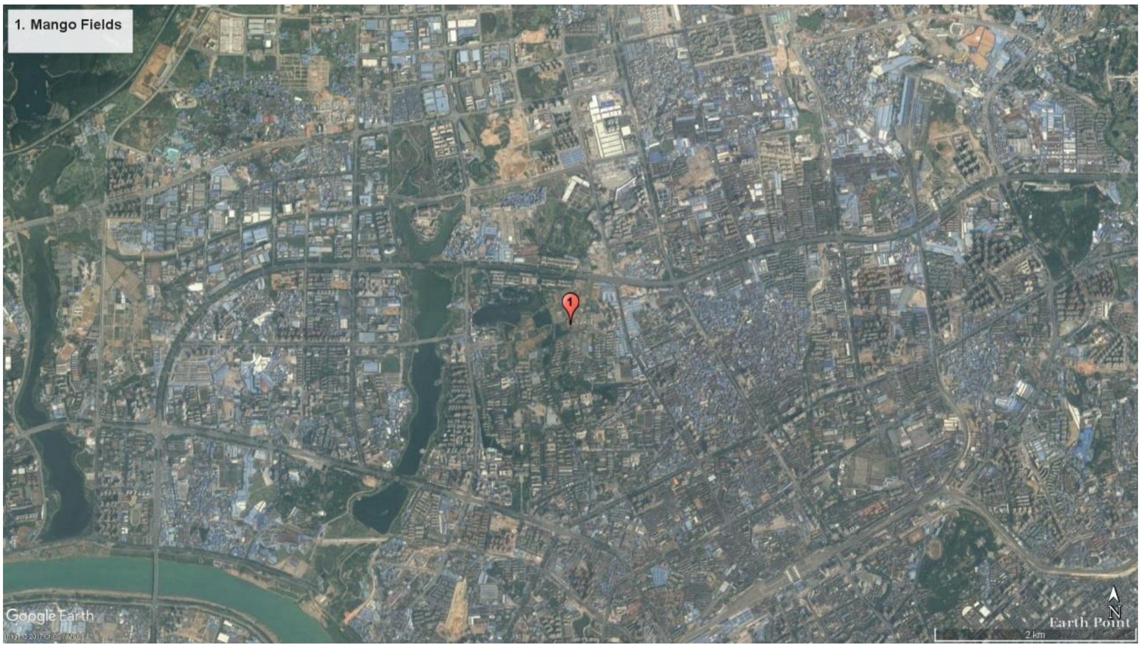

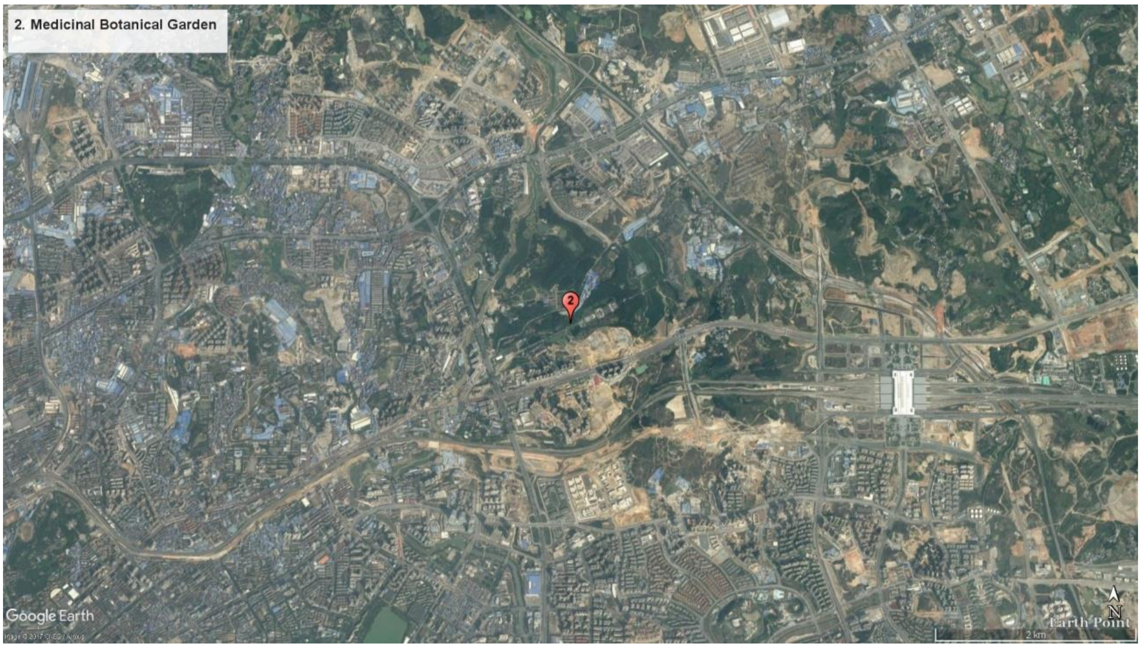

Rural locations

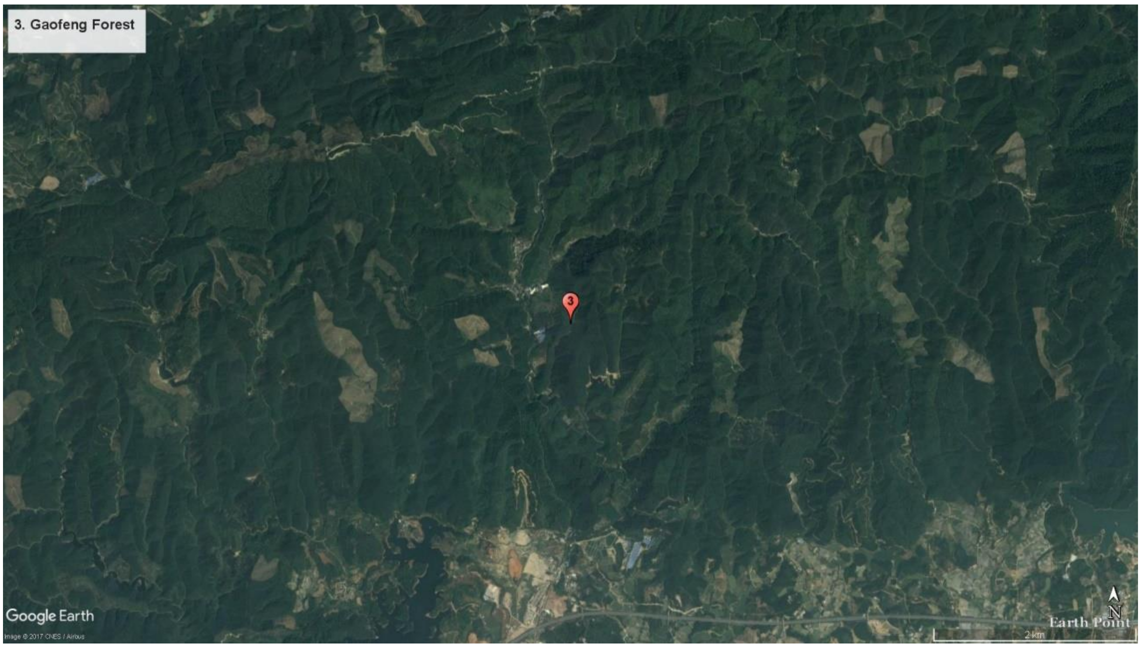

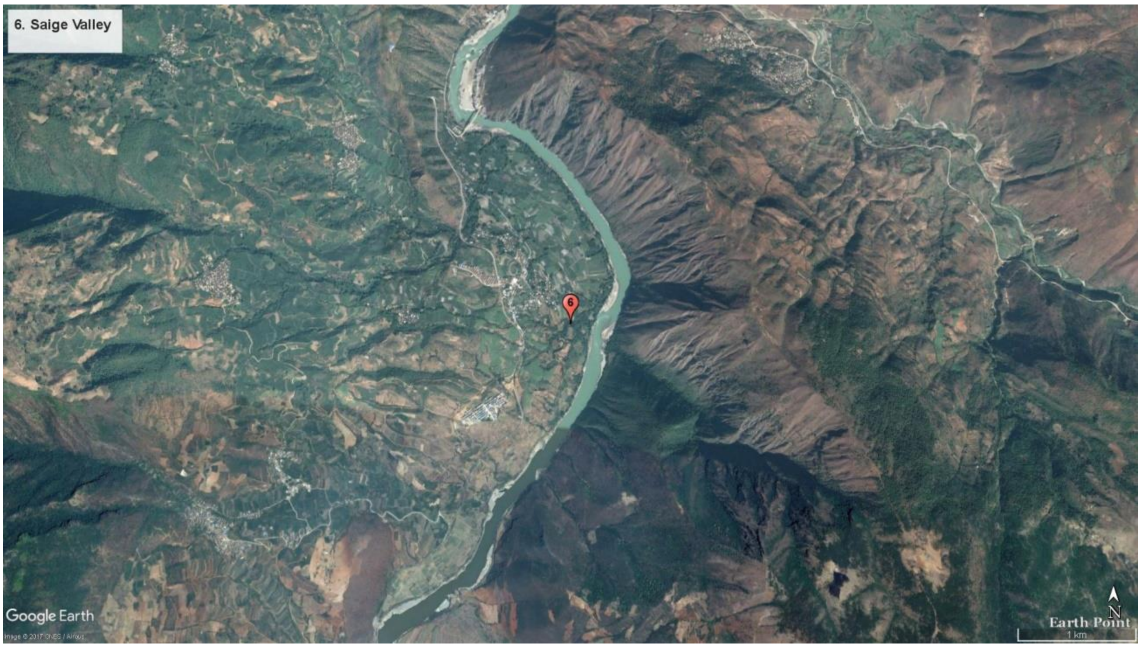

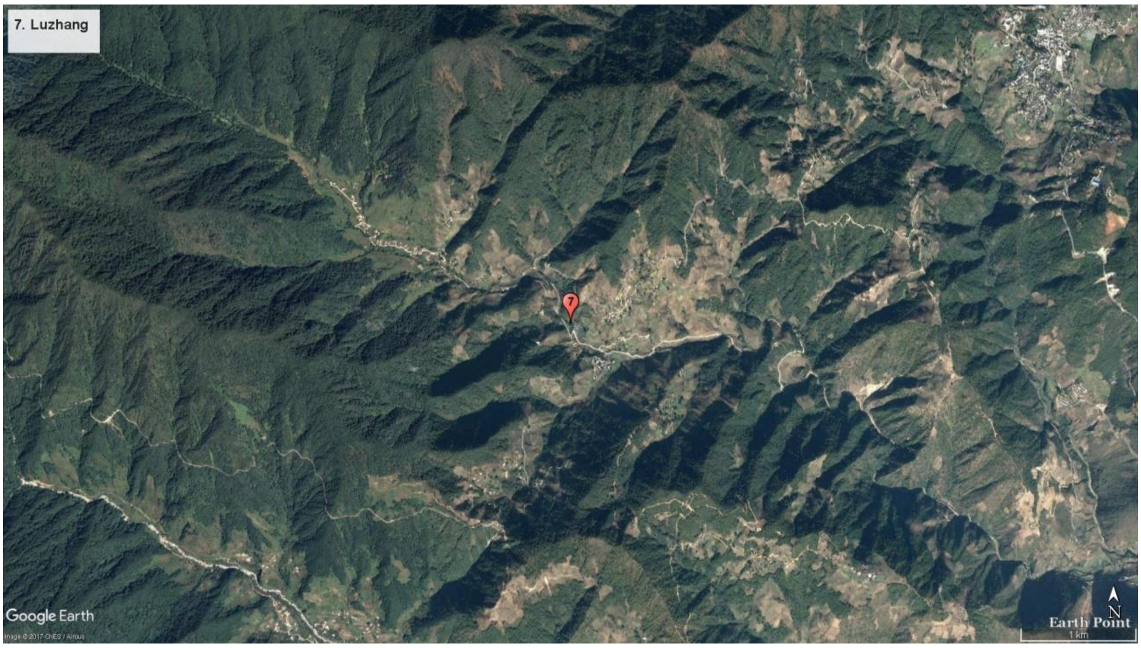

Natural locations

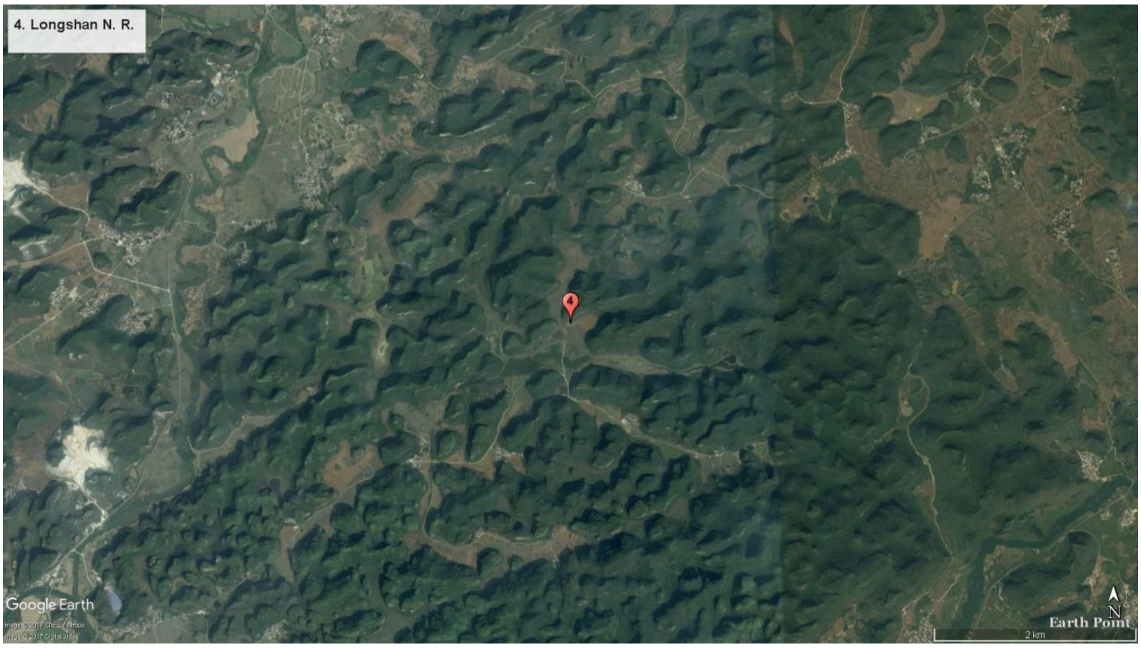

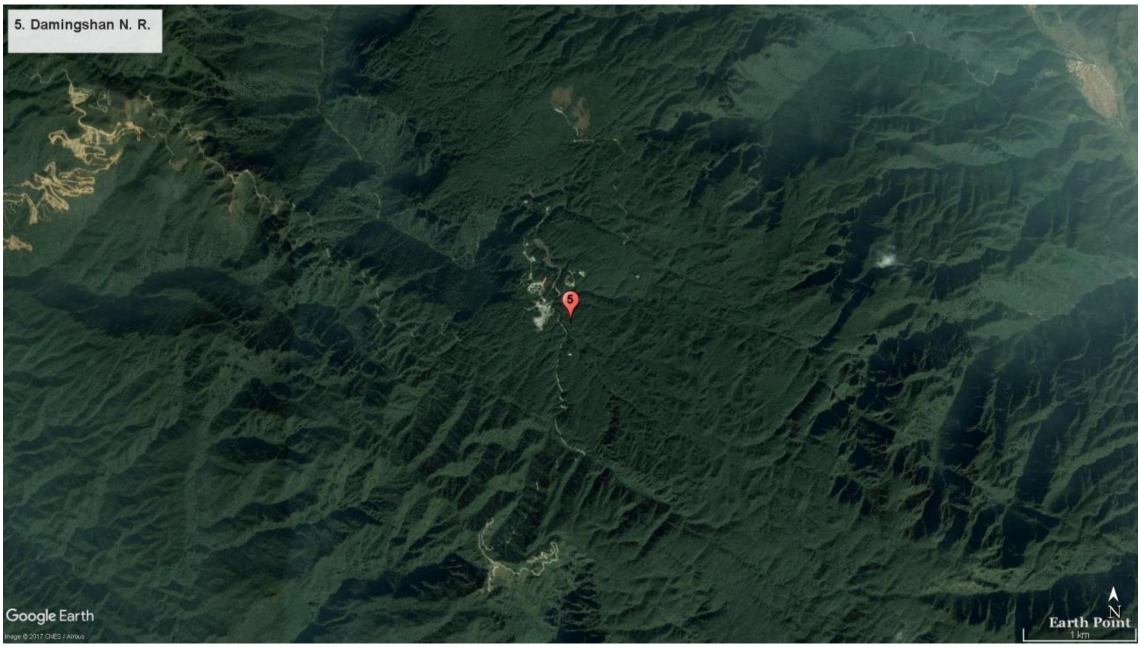
5. Damingshan N. R.
5
Google Earth
Earth Point
N
1 km

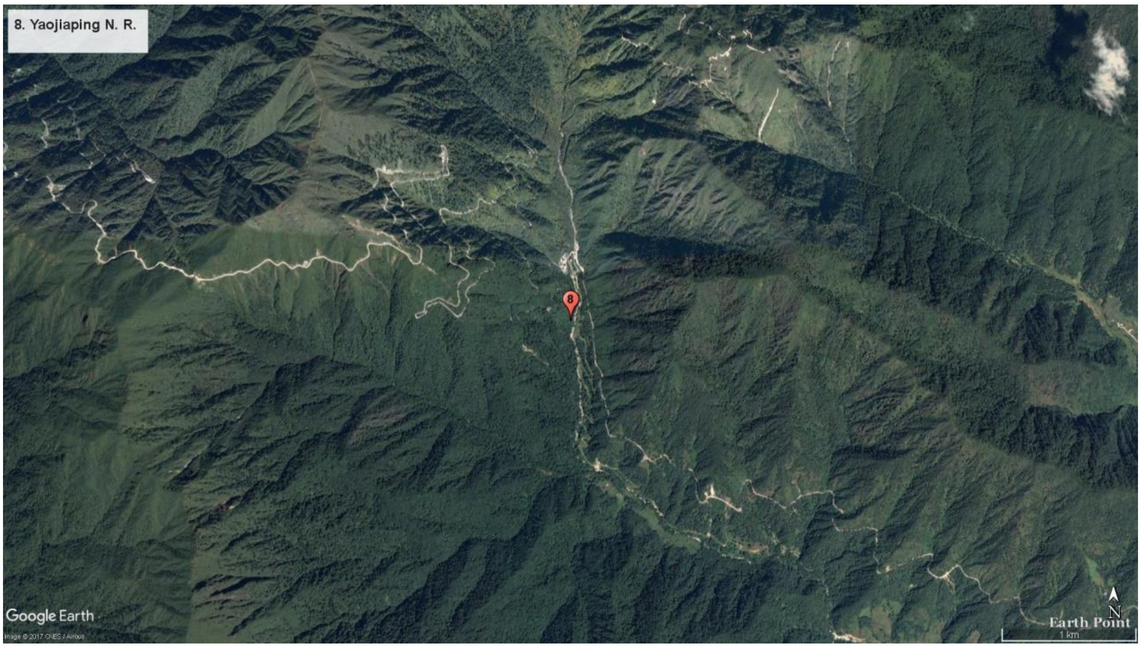
8. Yaojiaping N. R.
8
Google Earth
Earth Point
N
1 km
