# Supplementary material for: Breath rate of passerines across an urbanization gradient supports the pace‐of‐life hypothesis and suggests diet‐mediated responses to handling stress
Source: Ecol Evol. 2018 Aug 29;8(18):9526–35. doi: 10.1002/ece3.4460 (PMC6194294; doi:10.1002/ece3.4460)

Appendix 3 Plots of sex and age differences in breath rates. In this analysis, we only used species for which it was possible to determine sex and age based on external characteristics. Sex (male or female) was determined according to sexual colour dichromatism or the existence of brood patch. Age (juvenile or adult) was determined according to the presence of a moult limit in species that carry out a partial moult, or by the presence of nestling plumage characteristics. It can be appreciated that age and sex groups showed similar variability and strongly overlapped.

Sex differences

We used 100 females and 87 males from 57 species. Females mean ± SD = 67.53 ± 14.27. Males mean ± SD = 66.39 ± 14.92.

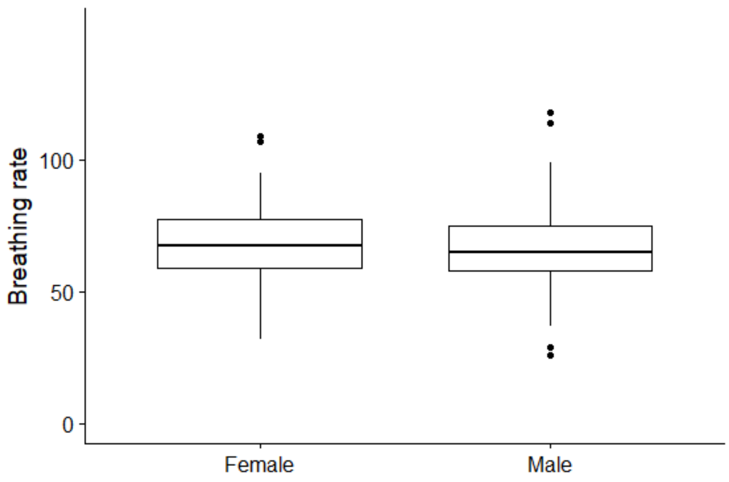

Age differences

We used 396 juveniles and 330 adults from 108 species. Juveniles mean ± SD = 69.23 ± 16.35. Adults mean ± SD = 67.85 ± 14.14.

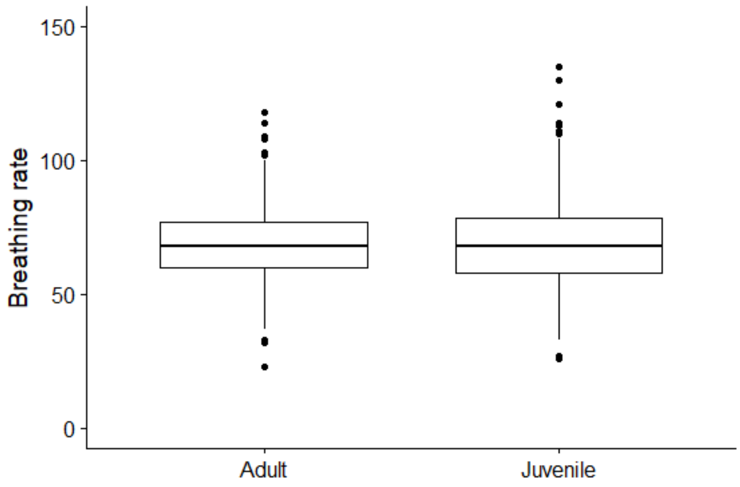

Supplement: Supplementary file 3 [file ECE3-8-9526-s003.pdf]
