# Supplementary material for: Breath rate of passerines across an urbanization gradient supports the pace‐of‐life hypothesis and suggests diet‐mediated responses to handling stress
Source: Ecol Evol. 2018 Aug 29;8(18):9526–35. doi: 10.1002/ece3.4460 (PMC6194294; doi:10.1002/ece3.4460)
Supplement: Supplementary file 4 [file ECE3-8-9526-s004.pdf]

Appendix 4 DIC and delta DIC (Δ DIC) values used to compare candidate models. Using Bayesian Phylogenetic Mixed Models (BPMM), we included mean STD BR as response variable, diet, habitat, elevation, migratory status, and body mass (log transformed) as fixed variables. We dummy coded diet and habitat as three-level variables, and each of the levels of these two groups were manually changed as references in candidate models.

**Candidate models**	**Habitat-reference**	**Diet-reference**	**DIC**	***Δ DIC***
Model 1	Natural	Omnivore	344.2198	8.227
Model 2	Natural	Herbivore	340.9976	5.005
Model 3	Natural	Insectivore	345.9087	9.916
Model 4	Rural	Omnivore	344.5313	8.539
**Model 5**	**Rural**	**Herbivore**	**335.9924**	**0.000**
Model 6	Rural	Insectivore	346.0058	10.013
Model 7	Urban	Omnivore	344.8677	8.875
Model 8	Urban	Herbivore	344.9652	8.973
Model 9	Urban	Insectivore	345.1199	9.128
